# Physiology

**Published:** 1880-03

**Authors:** 


### PHYSIOLOGY.

ON THE ELIMINATION OF PHOSPHORIC ACID IN HERBIVOROUS ANIMALS.—By Bertram, in *Zeitschrift f. Biol.*, Band. XIV, Heft. III, 1878. (*Revue des Sc. Méd.*, July, 1879.)

Whilst among carnivorous animals the greater part of the phosphoric acid taken into the system is eliminated by the urine, among the herbivorous animals it is excreted almost wholly by the intestines. Liebig attributes this fact to the existence in the urine of the herbivorous animals of large quantities of carbonates, and the consequent insolubility of the acid phosphates.

Bertram has made, relative to this subject, experiments on herbivorous animals and on man. He has established, in fact, that among the herbivorous animals the phosphoric acid is eliminated with the fæces and not with the urine; but when a considerable quantity of acid phosphate of potash is added to the normal food, phosphoric acid appears in the urine, and that even to a marked degree. This does not take place, however, if the acid combines with calcium. In fact, calcium is always deficient when the urine is abnormally charged with phosphoric acid, and further, the ingestion of heavy doses of calcium salts causes the disappearance of the phosphates. Magnesia does not exert a like action. It follows, then, that the absence of the acid phosphates in the urine of the herbivorous animals is due to the richness of the vegetables in calcium salts.

In man the citrate of potash diminishes the proportion of the acid phosphates to a small degree; the citrate of calcium to an extent much more marked. The elimination of the acid phosphates becomes still less when to the citrate of potash the bicarbonate of calcium is added.

NORMAL REACTION OF SWEAT.—(*Pflüger's Archiv*, xviii. p. 494; *Centralblatt f. die med. Wiss.*, May 17, 1879.)

Trümpy and Luchsinger have been recently engaged upon a series of investigations to decide whether human sweat normally possesses an acid reaction. Luchsinger observed some time since, in the course of his experiments as to the existence of a distinct sweat center, that the secretion obtained from the paws of cats was alkaline, and not, as usually stated, acid. The skin covering these parts contains no sebaceous but only sweat glands, and from this fact the authors draw the conclusion that the sweat is usually alkaline, and that its acidity is due to the admixture with the free fatty acids present in the secretion from the sebaceous glands. This conclusion has lately been verified in respect to man. If the skin be carefully washed, and a secretion of sweat be then produced, either by means of warm baths, or by the injection of one-tenth gram of hydrochlorate of pilocarpin hypodermically, the sweat is at first alkaline, though occasionally it is acid, becoming alkaline when the secretion has continued for some length of time. The sweat secreted from the palm of the hand is always acid, although true sebaceous glands are absent; but it becomes alkaline even here when the secretion is prolonged. The sweat glands appear to perform the function of sebaceous glands in this part. A comparison is drawn between glands on the palm of the hand and those found in the ear. Those glands, dependent upon degenerative processes, normally decline upon the induction of a free secretion of sweat.

